# Supplementary material for: Veterans Health Administration staff experiences with suicidal ideation screening and risk assessment in the context of COVID-19
Source: PLoS One. 2021 Dec 28;16(12):e0261921. doi: 10.1371/journal.pone.0261921 (PMC8714081; doi:10.1371/journal.pone.0261921)
Supplement: S1 Appendix — (DOCX) [file pone.0261921.s001.docx]

**Interview Guide: Primary Care Staff**

*The questions below are the general topic areas we will explore with primary care team members. These questions will be modified in light of what is learned and to fit the expertise/discipline of the interviewee.*

Opening: Thank you for taking time to meet with me today. My name is __________ and I am a part of the research team that is investigating aspects of VA’s new suicide risk screening initiative, VA Risk-ID. In this initiative, patients who screen positive for depression and PTSD are also screened for suicidal ideation, using a three-stage screening process: Patient Health Questionnaire item 9, or I-9, the Columbia-Suicide Severity Rate Scale (C-SSRS), and the Comprehensive Suicide Risk Evaluation (CSRE). We would like to know how this initiative has impacted your work and your relationships with your Veterans. We would also like to know your thoughts on ways to improve suicidal ideation screening and ways to enhance patient engagement in treatment after screening.

When we scheduled this interview, we went over an Information Sheet that explains the study in detail, the risk and benefits associated with the study, and sent you a copy via email. Did you have any questions about this at this time? We will be audio-recording our discussion today in order to generate transcripts for analysis. Please note that all efforts will be made to protect your privacy; any identifying information linking you to the content of what you share with us today will not be included in our transcripts. Do you have any questions before we begin?”

*So, to get started with the interview, we need to obtain your verbal consent for the recording. Is it okay with you for us to record this interview?* [IF YES, START RECORDER]

*I’ve started the recorder. Would you please state your name for the verbal consent process?*

*Do you consent to being recorded to participate in this study?*

*Great, thank you.*

**Section 1. Background and Screening Process**

SI SCREENING PROCESS

1. Generally, how prepared do you feel to ask about suicidal thoughts or discuss suicide risk with patients?
2. Thinking about the current suicide risk screening process, can you tell me a little bit about how the process goes in your work setting?
3. What are your facility’s expectations regarding screening?
4. What are your local team or clinic’s expectations regarding screening?

Probes:

- What is your role in suicidal ideation screening and assessment?
- Who are the other team members involved with suicidal ideation screening (including PC-MHI)?
- How does your team communicate with one another about results of screening?

STAFF MEMBER PERCEPTIONS

1. What do you think of the suicide risk screening process?

Probes:

- How do you think patients respond to the screening?
- What is it like for you to participate in screening?
- What are the positive aspects of screening?
- What aspects could be improved?
- What gaps are there in the suicidal ideation screening process? How might these be fixed?

**Section 2. Discussing the Screening Questions and Next Steps with Patient**

COMMUNICATION

1. What do you tell patients, if anything, about why screening is being done?
2. What do you tell patients, if anything, about what happens with the screen results?
3. How do you typically deliver the questions (e.g., exactly as written, in your own words, when during the session)?
4. Tell me how you communicate with other team members (if at all) during the screening process.

THERAPEUTIC ALLIANCE

1. What strategies do you use for building rapport with patients who you do not know very well and who require screening?
2. Do you think patients understand why they are being screened? (if not, do you ever explain it to them, and how?)

COLLABORATIVE PREPARATION FOR ACTION

1. How often do you work with patients to identify treatment options or goals after screening? What does that process look like?
2. How prepared do you feel to offer resources to patients who endorse suicidal thoughts? Do you feel adequate resources exist (both broadly and within your specific location)? What resources would you like to provide that you maybe don’t have access to now?
3. Do you suggest self-management resources to patients in response to screen results? If so, can you give me some examples?

**Section 3. Process Improvement and Future directions**

FINAL THOUGHTS

1. In your opinion, what responsibility does the clinician or staff member have when working with patients to prevent suicide?

Probe: Do you think that suicide is preventable? (ask if appropriate in the context)

1. How could suicide risk assessment be improved to make the Veteran’s experience better or to better support the Veteran’s engagement in treatment?

Probe:

- Are there any other approaches, besides asking these questions, that you think could help you evaluate suicide risk with your patients?
- Are there any potential negative impacts that could arise from these screening questions?

3. Is there anything else you would like to say about your experience with suicidal ideation screening that you have not had a chance to say?

Potential additional probes, if appropriate: How has the screening changed as a result of COVID-19? Any changes in frequency of use or process?

Do you have any personal experiences that you think affect your thoughts on screening for suicide risk?
