# Supplementary material for: Veterans Health Administration staff experiences with suicidal ideation screening and risk assessment in the context of COVID-19
Source: PLoS One. 2021 Dec 28;16(12):e0261921. doi: 10.1371/journal.pone.0261921 (PMC8714081; doi:10.1371/journal.pone.0261921)
Supplement: Attachment — Submitted filename: PLOS response to reviewers 11.9.21.docx [file pone.0261921.s002.docx]

Responses to Reviewers' comments:

Reviewer #1: Overall, this was a well-written and thoughtful manuscript reporting clinical impressions of the VA transition to remote administration of Risk ID in the early stages of the COVID crisis. My primary concerns are: 1) that some of the background information about VA practices is not included and is not easily accessible to readers outside of that system, and 2) use of neutral language. Please see comments below:

Author response: We will respond to specific comments where they occur below.

Materials and Methods:
The opening paragraph of the methods refers to two VA initiatives (VA CSRE; PC-MHI) without providing citations or links to any online documentation. This information should be easily accessible to non-VA affiliated readers who may not be able to properly contextualize the methods and results without supplementary information.

Author response: Thank you for pointing out this oversight. We have provided additional description and added a relevant citation.

Results
Language should be more neutral in places. Phrases like “deep concern” instead of just “concern” would be better. Though I agree with the sentiment, this statement from the Discussion read as a value judgement:

"First, overall, staff exhibited ingenuity and flexibility as they attempted to cope with rapidly changing circumstances—their clear dedication to care and to Veterans was evident."

Author response: We agree that some of the language holds some value judgement. We have edited the language to be more neutral.

Discussion

The diversity of expertise among participants and hospital size and setting was a strength of the paper, but greater consideration of how those differences aligned with themes is warranted. For example, there is some discussion at length about a shift away from having nurses administer Risk ID. Was this occurring at larger hospitals, rural hospitals, etc.? Though COVID has been challenging across levels of expertise, did baseline staff experience impact decisions to shift assessment assignment?

The discussion of changes in quality of care would be more useful if the results were contextualized in the hospital setting, e.g., were there particular types of hospitals, or staff makeup, that were more typical of hospitals that made the decision to not carry out parts or Risk ID?

Author Response: Thank you for the suggestion to better describe the contextual differences in the focal clinics. The study was not designed for, and our qualitative sample did not allow for, meaningful comparison between sites. We intentionally sought out variation across these characteristics to ensure broad representation of ideas and experiences, which can lead to better identification of key themes and hypotheses for later study (Sofaer, 1999 p. 1104). We did reexamine our data, and as expected, due to substantial variation across site and provider characteristics we were unable to detect clear patterns of association. We do note in the discussion (line 367) that sites that had existing virtual care prior to the pandemic might have had an easier time adjusting. Further, we highlight the limited contextual data as a limitation.

I agree with the passage below, but what kinds of evaluations do the authors recommend? As a closed health care system, there are some unique opportunities for addressing such questions at a depth that is more difficult to achieve when working in a majority private health insurance setting. It would be good if the authors made some more concrete recommendations.

"Others struggled considerably and further analyses of reasons for this variation has potential to yield important insights about factors influencing system-level crisis response."

In the discussion, the authors draw distinctions between hospitals who were, and were not, able to transition to virtual care, but the discussion of characteristics of both groups was shallow. I appreciate that this is a preliminary, hypothesis-generating piece, but I don't have a strong sense of where the next logical step in this line of research lies. How would qualitative work like yours be integrated into larger scale program evaluation efforts?

Author Response: We agree that we did not outline clear next steps for further work. Given that we did not have sufficient data to identify contextual differences between sites that adapted their SI protocols versus those that did not, further work that more precisely measures contextual variables is suggested. We have outlined further recommendations on line 396.

Reference

Sofaer, S. (1999). Qualitative methods: what are they and why use them?. Health services research, 34(5 Pt 2), 1101.
